# Tape-Strip Proteomic Profiling of Atopic Dermatitis on Dupilumab Identifies Minimally Invasive Biomarkers

**DOI:** 10.3389/fimmu.2020.01768

**Published:** 2020-08-06

**Authors:** Helen He, Caroline M. Olesen, Ana B. Pavel, Maja-Lisa Clausen, Jianni Wu, Yeriel Estrada, Ning Zhang, Tove Agner, Emma Guttman-Yassky

**Affiliations:** ^1^Department of Dermatology, Icahn School of Medicine at Mount Sinai, New York, NY, United States; ^2^Department of Dermatology, Bispebjerg University Hospital, Copenhagen, Denmark

**Keywords:** atopic dermatitis, dupilumab, tape-strips, olink, proteomics, targeted therapeutics, atherosclerosis, cardiovascular disease

## Abstract

Tape-stripping is a minimally invasive approach for skin sampling that captures the cutaneous immune/barrier abnormalities in atopic dermatitis (AD). However, tape-strips have not been used to evaluate molecular changes with therapeutic targeting. In this study, we sought to characterize the proteomic signature of tape-strips from AD patients, before and after dupilumab therapy. Twenty-six AD patients were treated with every-other-week dupilumab 300 mg for 16 weeks. Tape-strips from lesional and non-lesional skin were collected before and after treatment, and analyzed with the Olink proteomic assay. Using criteria of fold-change>1.5 and FDR < 0.05, 136 proteins significantly decreased after dupilumab treatment, corresponding to an overall mean improvement of 66.2% in the lesional vs. non-lesional AD proteome. Significant decreases after dupilumab were observed in immune markers related to general inflammation (MMP12), Th2 (CCL13/CCL17), Th17/Th22 (IL-12B, CXCL1, S100A12), and innate immunity (IL-6, IL-8, IL-17C), while the Th1 chemokines CXCL9/CXCL10 remained elevated. Proteins related to atherosclerosis/cardiovascular risk (e.g., SELE/E-selectin, IGFBP7, CHIT1/ chitotriosidase-1, AXL) also significantly decreased after treatment. Dupilumab therapy suppressed AD-related immune biomarkers and atherosclerosis/cardiovascular risk proteins. Tape-strip proteomics may be useful for monitoring therapeutic response in real-life settings, clinical trials, and longitudinal studies for AD and beyond.

## Introduction

Atopic dermatitis (AD) is a common inflammatory skin disease, characterized by epidermal dysfunction and T-helper 2 (Th2)/Th22-predominant inflammation, with variable increases in Th1 and Th17 (1–11). Dupilumab, a monoclonal antibody that targets IL-4Rα, inhibiting IL-4/IL-13 signaling, has demonstrated both clinical efficacy and modulation of Th2 cytokines (e.g., IL-13, CCL17, CCL18, CCL26) and other inflammatory mediators in skin from AD patients (1, 12–14). However, these molecular studies used biopsies, which are associated with pain, scarring, and increased risk of infection. Hence, there is a need for less invasive skin sampling techniques for monitoring therapeutic response in real-life, clinical trials, and longitudinal studies.

Tape-stripping, a minimally invasive approach that samples the stratum corneum and upper stratum granulosum (15, 16), has been used to evaluate AD skin biomarkers, almost exclusively in untreated patients (16–26). Koppes et al. reported decreases in Th2 markers (e.g., IL-4, IL-13, CCL22) after local application of emollient to the skin (27). However, to our knowledge, tape-strips have not yet been utilized to assess changes in the AD cutaneous profile in response to systemic targeted therapeutics like dupilumab.

Recently, we used the Olink multiplex assay to characterize the cutaneous proteomic profile of a large panel of ~350 inflammatory proteins in skin biopsies from untreated moderate-to-severe AD patients, requiring only 10 ng of tissue (28). Thus, far, this platform has not been used to analyze tape-strips, or to measure therapeutic response in inflammatory skin conditions.

In this real-life study, we used Olink to define the proteomic signature of 26 moderate-to-severe AD patients in tape-strips collected from lesional/non-lesional skin, before and after 16 weeks of dupilumab treatment. Dupilumab therapy resulted in significant reductions in products of pathogenic immune axes (e.g., Th2, Th17) and in markers that have been implicated as prognostic or therapeutic targets of atherosclerosis and cardiovascular disease (CVD).

## Methods

### Study Population

Twenty-six Caucasian patients with atopic dermatitis (AD) [four female/22 male; age range, 18–65 years [mean, 41.2 years]; mean EASI 19.2] initiating treatment with dupilumab at the Department of Dermatology, Bispebjerg Hospital, Denmark, between March 2018 and June 2019 were enrolled under institutional review board-approved protocols (Table 1). The patients were treated with subcutaneous injections of 300 mg dupilumab every 2 weeks for 16 weeks, after a loading dose of 600 mg (label use). To be considered for dupilumab treatment, patients had to meet the following criteria: AD according to the UK-criteria, age ≥ 18 years, and history of failed treatment with two or more traditional systemic immunosuppressive therapies. Of note, all enrolled patients reported no history of other known inflammatory skin (e.g., psoriasis), autoimmune (e.g., rheumatoid arthritis), or systemic disease (e.g., cardiovascular disease, coronary, or carotid artherosclerosis). The use of topical anti-inflammatory treatments and emollients was prohibited seven days and 24 h prior to each evaluation, respectively. Twenty-three patients had not received treatment with phototherapy or systemic immunosuppressive drugs 4 weeks prior to dupilumab treatment, while two patients had been treated with prednisolone and one with methotrexate within 4 weeks of the first visit.

**Table 1:** Demographic Table.

**Characteristic**	**Atopic dermatitis: Baseline (*n = 26*)**	**Atopic dermatitis: Post-treatment**	**P-value**
Age, mean ±SD	41.2 ± 11.0	–	
Gender			
Female	4	–	
Male	22		
EASI, mean ±SD	19.2 ± 8.3	5.3 ± 5.5	4.12E-09

*EASI, Eczema Area and Severity Index; SD, Standard deviation*.

### Tape-Strip Collection and Protein Extraction

Patients were evaluated before and 16 weeks after initiating treatment with dupilumab. At each visit, thirty consecutive tape-strips (D-Squame 3.8 cm^2^, CuDerm) were collected from representative lesions and non-lesional skin on the upper or lower extremities, and were serially labeled. Each tape was pressed against skin for 10 s with a standardized pressure (225 gr/cm^2^), before being stripped from the skin. Tapes number 3 and 18 were pooled together and used for subsequent protein extraction. For each sample, tapes 3 and 18 were cut into quarters and placed in 8 tubes. 300 μL of Ripa buffer were added to the first quarter, followed by 15 min sonification in iced water (0–4°C) in an ultrasonic bath. The extract was then transferred to the next quarter followed by 15 min sonification. This was repeated a total of eight times for each sample.

### Olink Proteomic Analysis

For each sample, 10 ng of protein was lysed in 90 μL of protein extract, and was analyzed using the high-throughput proteomic OLINK Proseek® multiplex assay (a proximity extension assay using oligonucleotide-labeled antibody probe pairs) (29). Pre-selected Inflammation and Cardiovascular disease (CVDII/III) panels were assessed, as previously described, comprising a total of 353 proteins (28, 30).

### Statistical Analysis

A power calculation from a published Olink study that evaluated patients with AD (28) showed that a sample size of 25 subjects would allow us to detect differences in lesional compared to non-lesional skin in Th2 (CCL13, CCL17), Th1 (CXCL9, CXCL10), and atherosclerosis/cardiovascular risk markers (SELE/E-selectin, SELP/P-selectin, RETN/resistin) with more than 90% power (at significance level 0.05). Statistical analyses were performed using R software (www.R-project.org) and packages available through Bioconductor (www.bioconductor.org). Fold-changes were estimated, and hypothesis testing was conducted using contrasts under the general framework for linear models in the *limma* package. *P*-values were adjusted for multiple hypotheses using the Benjamini-Hochberg procedure, controlling for false discovery rate (FDR). Proteins with fold-change >1.5 and FDR < 0.05 were considered differentially expressed. Unsupervised hierarchical clustering of mean expression profiles was performed with Euclidean distance and a McQuitty agglomeration scheme, and represented by a heatmap and accompanying dendrogram. Previously published protein sets of Th1, Th2, Th17, and atherosclerosis/cardiovascular risk proteins were quantified by gene-set variation analysis (*gsva* package), and expressed as z-scores (1, 28). Spearman correlations were used to assess correlations between percent improvement of clinical severity scores and percent change in protein expression levels.

## Results

### Overall Clinical Improvement and Proteomic Changes in AD Patients After Dupilumab Therapy

Twenty-six adults with moderate-to-severe AD were included in this study (mean age 41.2 years, four female/22 male, mean EASI: 19.2) (Table 1). Overall, AD patients experienced significant clinical improvement, with mean percentage reduction in EASI score of 72.4% (*P* < 0.001) after 16 weeks of dupilumab therapy, consistent with previously reported efficacy of dupilumab in AD (Table 1) (1).

Tape-stripped lesional and non-lesional skin samples were analyzed with the Olink proteomic assay before and after treatment, measuring 353 inflammatory proteins. Using criteria of fold-change > 1.5 and FDR < 0.05, we identified 184 up-regulated proteins in lesional vs. non-lesional AD at baseline, of which 136 proteins significantly decreased after dupilumab therapy in lesional and/or non-lesional skin (Table S1). Dupilumab therapy induced an overall mean improvement of 66.2% in lesional vs. non-lesional proteome.

### Dupilumab Suppressed Activation of Th2 and Th17/Th22, but Not Th1 Immune Pathways

**Figure 1:**
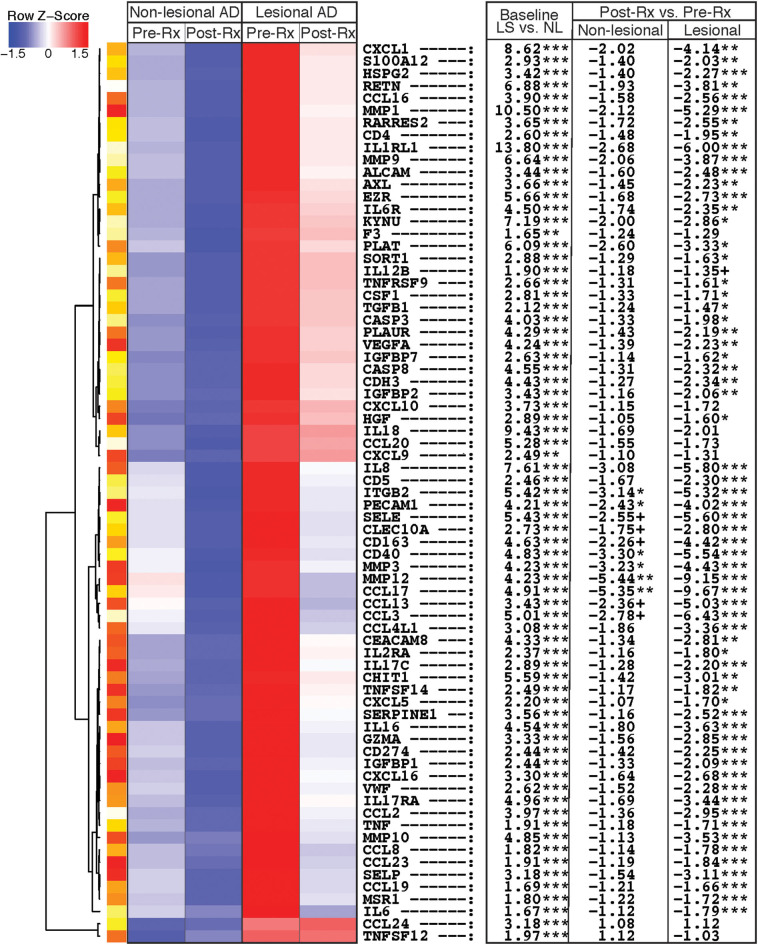
Heatmap of immune and atherosclerosis/cardiovascular proteins. Heatmap of immune and atherosclerosis and cardiovascular signaling proteins that are differentially expressed in tape-stripped skin of atopic dermatitis patients before and after dupilumab therapy, using the criteria of fold-change >1.5 and false discovery rate <0.05. Proteins are ordered by unsupervised hierarchical clustering, as represented by dendrogram on the left. The table on the right shows fold-changes in lesional vs. non-lesional tape-strips at baseline (LS vs. NL), and post- vs. pre-treatment with dupilumab (Post-Rx vs. Pre-Rx) for both non-lesional and lesional skin. LS, Lesional; NL, Non-lesional; Rx, Treatment.***FDR < 0.001, **FDR < 0.01, *FDR < 0.05, +FDR < 0.1.

Proteins that showed the greatest differential expression at baseline or with treatment, and are known to be part of immune or atherosclerosis/cardiovascular signaling pathways (1, 28, 31), are depicted in an unsupervised hierarchical clustering heatmap (Figure 1). Several cellular markers of immune cell infiltration were elevated in lesional vs. non-lesional skin at baseline, and significantly decreased in lesional skin after dupilumab. These included markers of macrophages (CSF1, CD163), DCs (CLEC10A, CD40, TNF), and T-cells (CD4, CD5, GZMA) (Figure 1, Table S1).

Immune proteins related to general inflammation (MMP12), T-cell activation/migration (IL-2RA, CCL19, TNFRSF9, EZR), Th2 (CCL13, CCL17, CCL23, IL-1RL1/ST2/IL-33R), Th17/Th22 (IL-12B, IL-17RA, CXCL1, KYNU, S100A12), and innate immunity (IL-6, IL-8, IL-17C, IL-6R) similarly displayed significant increases at baseline and significant reductions in lesions after dupilumab treatment (Figure 1, Table S1). CCL2 and CCL3, which signal via both Th1 and Th2 pathways, were up-regulated at baseline and significantly suppressed after dupilumab in tape-stripped lesions, while the Th1 chemokines CXCL9 and CXCL10 were initially up-regulated in lesional vs. non-lesional tape-stripped skin at baseline, but did not change significantly with treatment.

Few immune markers demonstrated significant reductions with dupilumab therapy in tape-strips collected from non-lesional skin. These included the DC marker CD40, and Th2 chemokine CCL17, with a trend toward significance (FDR < 0.1) for macrophage/DC markers (CD163, CLEC10A), Th2 chemokine CCL13, and Th1/Th2 chemokine CCL3 (Figure 1, Table S1).

### Dupilumab Decreased Inflammatory Proteins Related to Atherosclerosis Signaling and Cardiovascular Risk

Numerous proteins that have been linked to atherosclerosis and CVD showed up-regulation in baseline tape-stripped lesional skin, with significant suppression after dupilumab treatment (Figure 1, Table S1). These included pro-atherogenic cytokines (IL-16, CCL2, CCL8), mediators of vascular adhesion and/or angiogenesis (SELE/E-selectin, SELP/P-selectin, VEGFA, HSPG2/perlecan, HGF/hepatocyte growth factor, PECAM1/platelet endothelial cell adhesion molecule-1, VWF/von Willebrand factor), and matrix metalloproteinases (MMPs) involved in atherosclerotic tissue remodeling (MMP1, MMP3, MMP9, MMP10). PECAM1 and MMP3 also demonstrated significant reductions with therapy in non-lesional skin, with a trend toward significance (FDR < 0.1) for SELE/E-selectin.

Dupilumab significantly decreased levels of proteins previously described as prognostic markers or therapeutic targets of cardiovascular morbidity/mortality and cardiac injury in lesional skin, including TNFSF14/LIGHT, ALCAM/activated leukocyte cell adhesion molecule, IGFBP1/insulin-like growth factor binding protein-1, IGFBP2, IGFBP7, SORT1/sortilin, SERPINE1/plasminogen-activator inhibitor type-1 (PAI-1), CHIT1/chitotriosidase-1, PLAUR/uPAR, AXL/tyrosine-protein kinase receptor UFO, and RETN/resistin (Figure 1, Table S1). Dupilumab also suppressed markers of cell-cell adhesion (CDH3/cadherin-3), apoptosis (CASP3/caspase-3, CASP8/caspase-8), and negative immune regulation (CD274/PD-L1).

### Pathway Analysis Supports Protein Differential Expression by Olink

**Figure 2:**
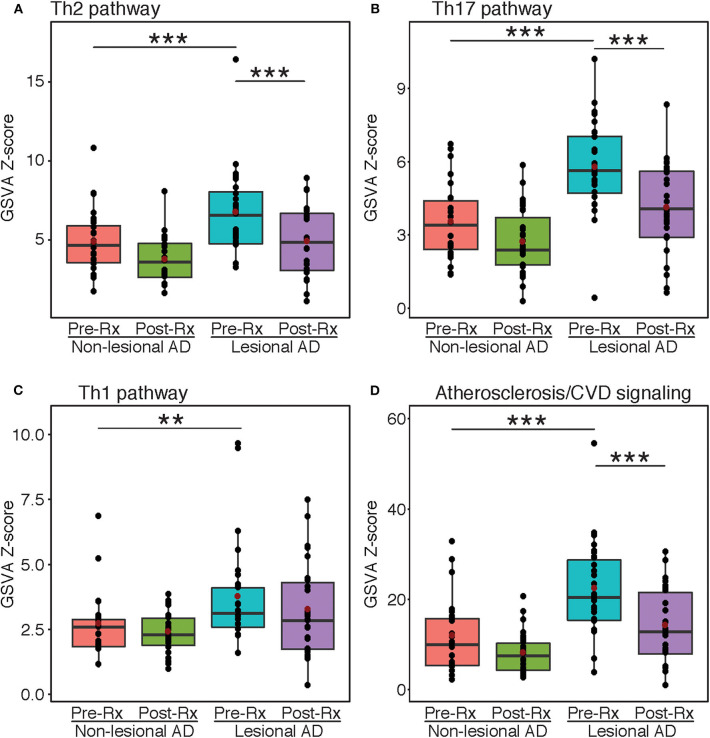
Gene-set variation analysis. Mean z-scores of the Th2 **(A)**, Th17 **(B)**, Th1 **(C)**, and atherosclerosis/cardiovascular risk **(D)** pathways in atopic dermatitis lesional and non-lesional tape-strips before and after dupilumb therapy, expressed as a boxplot. Red dots indicate mean values. Asterisks indicate significance between respective groups. AD, Atopic dermatitis; GSVA, Gene-set variation analysis; Rx, Treatment. ***FDR < 0.001, **FDR < 0.01.

Gene-set variation analyses were performed using immune and atherosclerosis/cardiovascular-related pathways that were previously published (Figure 2) (1, 28, 31). The Th2, Th17, and Th1 pathways were all highly enriched at baseline in lesional vs. non-lesional skin (FDR < 0.01) (Figures 2A–C). However, only Th2 and Th17, but not Th1, showed significant reductions after dupilumab treatment (FDR < 0.001). For Th1, the lack of significant difference between the non-lesional group and post-treatment lesional group is likely due to the inclusion of markers that are co-regulated by other immune axes (e.g., CCL2 and CCL3 are co-regulated by Th1 and Th2). Dupilumab also significantly inhibited the pathway of atherosclerosis/cardiovascular risk-related proteins (FDR < 0.001) (Figure 2D).

### Correlation Analyses Showed Strong Associations Between Lesional Protein Expression Changes and Clinical Improvement

**Figure 3:**
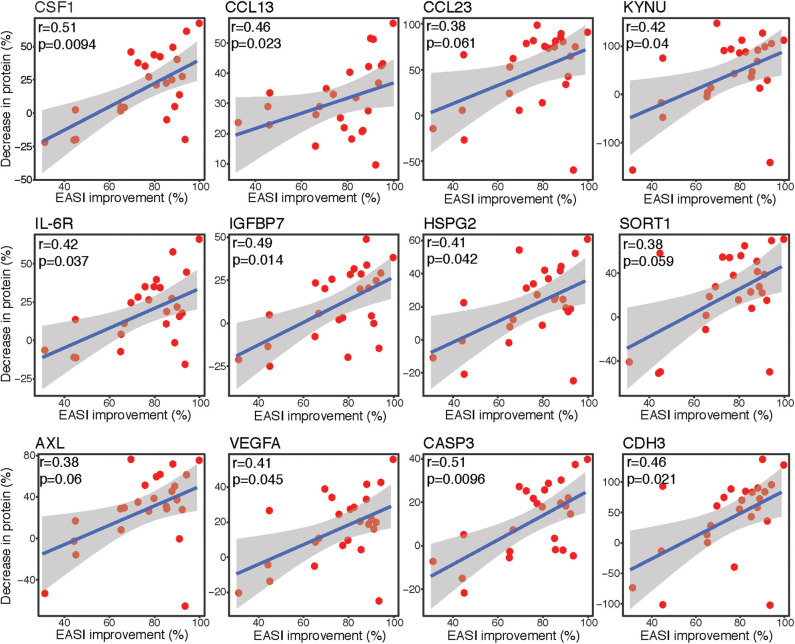
Correlation plots. Spearman correlation scatter plots [linear regression [blue line] with its confidence interval [gray area]] for percent improvement in EASI score before and after dupilumab vs. percent decrease in protein expression levels, as measured by Olink. Upper left corner with r, Spearman correlation coefficient and p, associated *P*-value. EASI, Eczema Area and Severity Index.

Spearman correlation analyses were performed to assess the associations between differences in protein expression levels in lesional tape-stripped skin and clinical severity, as measured by EASI. The differentially expressed proteins with the strongest correlations between percent decrease in expression level and percent improvement in EASI are depicted in Figure 3. The percent improvement in EASI strongly correlated with the percent decrease in markers of macrophages (CSF1), Th2 (CCL13, CCL23), Th17 (KYNU), and innate immunity (IL-6R) (*r* > 0.35, *P* < 0.1). Similar correlations were observed for factors implicated in cardiovascular mortality and morbidity (IGFBP7, HSPG2/perlecan, SORT1/sortilin, AXL), as well as mediators of angiogenesis (VEGFA), apoptosis (CASP3), and cell-cell adhesion (CDH3) (*r* > 0.35, *P* < 0.1).

## Discussion

In this first tape-strip proteomic profiling study that evaluates changes with dupilumab therapy, we discovered that dupilumab therapy resulted in alterations in important immunologic and atherosclerosis/cardiovascular proteins, many of which were also found in skin biopsies to be elevated at baseline or modulated by dupilumab therapy (1, 6, 12, 28, 32). This current study provides several novel findings. First, while tape-strips have been used to map the lesional and non-lesional AD cutaneous profiles at baseline (16, 17), tape-strips have never been utilized to track molecular response to targeted treatment. Furthermore, the Olink proteomic assay, recently used to profile skin biopsies (28), has not yet been applied to analysis of tape-strips. Compared to mRNA profiling, Olink enables quantification of specific proteins related to CVD, and perhaps also allows for detection of greater, more significant differences between lesional and non-lesional AD in tape-strips (17). Finally, this is the first cutaneous study characterizing changes in the AD proteomic profile in response to treatment.

Decreases in markers of T-cells/T-cell activation (CD4, IL-2RA), macrophages (CSF1, CD163), and DCs (CLEC10A, CD40) were observed in AD lesions after dupilumab therapy. These effects were not limited to lesions, as dupilumab also down-regulated expression of CD163, CLEC10A, and CD40 in clinically unaffected skin. IL-4 and IL-13 are proposed to have a role in directing maturation/activation of macrophages and DCs, including atopic DCs (33, 34). Dupilumab likely inhibits these pathways, resulting in diminished infiltration by immune cells in lesional and non-lesional skin.

Predictably, dupilumab induced significant reductions in key Th2 markers, including CCL13, CCL17, and IL-1RL1/ST2/IL-33R, consistent with its mechanism of action in inhibiting the Th2 axis via IL-4/IL-13 signaling. IL-1RL1, in particular, is also a prognostic factor for heart failure (HF) and myocardial infarction (MI) (35), forming a bridge between Th2 activation/allergy sensitization and CVD. Several Th17 products (IL-12B, CXCL1, KYNU) also decreased after dupilumab therapy, supporting the role of IL-4 in regulating Th17, suggesting that downstream signaling pathways via IL-4/IL-13 also influence the Th17 axis, possibly related to its regulation of DC differentiation. In contrast, the Th1/IFNG-related chemokines CXCL9 and CXCL10 remained unchanged, suggesting that dupilumab has a minimal effect on the Th1 pathway. Observed decreases in the Th1 chemokines CCL2 and CCL3 are likely due to their co-regulation by Th2 (36, 37). The Th2 and Th1 axes are known to have reciprocal effects on each other (38, 39), so Th2 inhibition with dupilumab may paradoxically relieve suppression of Th1. Notably, our proteomic data in tape-strips are consistent with mRNA profiling in skin biopsies, where dupilumab also reduced cellular T-cell/DC infiltrates and Th2/Th17 products, without appreciable Th1 modulation (1, 12).

Previous Olink studies have highlighted increased levels of atherosclerosis and cardiovascular-risk proteins in AD compared to healthy individuals in both blood and skin (28, 30, 31, 40). From these studies, we postulated that these inflammatory proteins originate in the skin, but spill into the blood, where they induce pro-inflammatory effects systemically. However, it has yet to be determined whether these abnormalities are affected by dupilumab or any other treatment. Here, we present data indicating that many atherosclerosis and cardiovascular risk-related proteins that were found to be up-regulated in other Olink studies (e.g., SELE/E-selectin, TNFSF14/LIGHT, VEGFA, RETN/resistin, MMPs) are in fact suppressed by dupilumab in tape-stripped skin lesions (28, 30, 31, 40).

We further identified additional proteins that may be implicated in cardiovascular mortality or morbidity, but have not been linked to AD in the past. For example, IGFBP7 was recently found to be more elevated in patients with MI, and may be a good biomarker of coronary artery disease (CAD) occurrence (41). High serum levels of ALCAM were independently associated with an increased risk of cardiovascular death in acute coronary syndrome (42). CHIT1/chitotriosidase-1, secreted by activated macrophages, is expressed at higher levels in patients with atherosclerosis in a severity-dependent manner (43), and may be a predictor of endothelial dysfunction and insulin resistance in type 2 diabetes (44). PLAUR/uPAR, which is expressed by vascular endothelial cells and is involved in atherosclerotic plaque destabilization, outperformed CRP in predicting CVD (45, 46). High serum levels of PAI-1, encoded by SERPINE1, suppresses fibrinolysis and may thus increase the risk of CAD (47), and is also an independent risk factor for reinfarction in patients who survived an early MI (48). Finally, AXL, which may be a mediator of myocardial damage, had higher expression in patients with HF, and may be associated with worse HF prognosis (49). Taken together, dupilumab modified lesional skin expression of various prognostic markers and potential therapeutic targets of CVD, highlighting these as potential biomarkers of therapeutic response in AD, requiring further exploration in future studies. Furthermore, these decreases in skin may precede longer-term effects in AD patients, like decreased risk of future cardiac complications, also meriting further investigation with larger longitudinal studies. If these markers in skin indeed have prognostic value for systemic inflammation and CVD burden, the fact that they are quantifiable by minimally invasive tape-strips would have vast applications in diagnosis and clinical evaluation of not just AD, but other inflammatory skin and systemic diseases.

This study had a few limitations. Patients were only followed up at 16 weeks, while longer-term follow-up would be beneficial to assess the persistence of proteomic changes induced by dupilumab. Additionally, protein quantification in this study was limited to 353 proteins. The investigated panels did not include important Th22 markers like IL-22, therefore precluding careful examination of the Th22 axis. We also did not assess a placebo group, since our study simulated a real-life experience. Finally, our sample size, while relatively small, is powered to detect significant differences in important disease-related markers. Validation of these findings with a larger cohort in the future would be beneficial.

In summary, we present the first study investigating changes in the proteomic signature of tape-strips collected from lesional/non-lesional AD skin before and after dupilumab therapy. Our data showed treatment-related suppression of key immune and atherosclerosis/cardiovascular risk proteins, further correlating with clinical improvement. These data emphasize the potential utility of tape-strip proteomic profiling for tracking biomarkers of therapeutic response in real-life settings as well as clinical trials and longitudinal studies of AD and beyond.

## Data Availability Statement

The raw data supporting the conclusions of this article will be made available by the authors, without undue reservation.

## Ethics Statement

The studies involving human participants were reviewed and approved by Local IRB approval in Icahn School of Medicine and Bispebjerg University Hospital. The patients/participants provided their written informed consent to participate in this study.

## Author Contributions

HH, CO, AP, TA, and EG-Y designed the research study. CO, M-LC, and TA recruited and collected tape-strips from patients. CO, YE, and NZ extracted protein for proteomic analysis. HH and AP performed statistical analyses. HH, CO, JW, TA, and EG-Y wrote the manuscript. All authors critically revised the manuscript and approved its final form.

## Conflict of Interest

EG-Y was an employee of Mount Sinai and has received research funds (grants paid to the institution) from: Abbvie, Celgene, Eli Lilly, Janssen, Medimmune/Astra Zeneca, Novartis, Pfizer, Regeneron, Vitae, Glenmark, Galderma, Asana, Innovaderm, Dermira, UCB. EG-Y was also a consultant for Sanofi Aventis, Regeneron, Stiefel/GlaxoSmithKline, MedImmune, Celgene, Anacor, AnaptysBio, Dermira, Galderma, Glenmark, Novartis, Pfizer, Vitae, Leo Pharma, Abbvie, Eli Lilly, Kyowa, Mitsubishi Tanabe, Asana Biosciences, and Promius. TA was an advisor/investigator or speaker for Pfizer Inc., AbbVie, Eli Lilly, LEO Pharma, Regeneron, and Sanofi-Genzyme. The remaining authors declare that the research was conducted in the absence of any commercial or financial relationships that could be construed as a potential conflict of interest.

## Abbreviations

AD: Atopic dermatitis
CAD: Coronary artery disease
CCL: C-C motif chemokine ligand
CVD: Cardiovascular disease
CXCL: C-X-C motif chemokine ligand
DC: Dendritic cell
EASI: Eczema Area and Severity Index
FDR: False discovery rate
HF: Heart failure
IL: Interleukin
MI: Myocardial infarction
Th: T-helper.

## References

[1] Guttman-Yassky E Bissonnette R Ungar B Suarez-Farinas M Ardeleanu M Esaki H. Dupilumab progressively improves systemic and cutaneous abnormalities in patients with atopic dermatitis. J Allergy Clin Immunol. (2019) 143:155–72. 10.1016/j.jaci.2018.08.022 30194992

[2] Agrawal R Woodfolk JA. Skin barrier defects in atopic dermatitis. Curr Allergy Asthma Rep. (2014) 14:433. 10.1007/s11882-014-0433-9 24633617 PMC4034059

[3] Czarnowicki T Krueger JG Guttman-Yassky E. Novel concepts of prevention and treatment of atopic dermatitis through barrier and immune manipulations with implications for the atopic march. J Allergy Clin Immunol. (2017) 139:1723–34. 10.1016/j.jaci.2017.04.004 28583445

[4] Gittler JK Shemer A Suárez-Fariñas M Fuentes-Duculan J Gulewicz KJ Wang CQ. Progressive activation of T(H)2/T(H)22 cytokines and selective epidermal proteins characterizes acute and chronic atopic dermatitis. J Allergy Clin Immunol. (2012) 130:1344–54. 10.1016/j.jaci.2012.07.012 22951056 PMC3991245

[5] Boguniewicz M Leung DY. Atopic dermatitis: a disease of altered skin barrier and immune dysregulation. Immunol Rev. (2011) 242:233–46. 10.1111/j.1600-065X.2011.01027.x 21682749 PMC3122139

[6] Suarez-Farinas M Ungar B Correa Da Rosa J Ewald DA Rozenblit M Gonzalez J. RNA sequencing atopic dermatitis transcriptome profiling provides insights into novel disease mechanisms with potential therapeutic implications. J Allergy Clin Immunol. (2015) 135:1218–27. 10.1016/j.jaci.2015.03.003 25840722

[7] Broccardo CJ Mahaffey S Schwarz J Wruck L David G Schlievert PM. Comparative proteomic profiling of patients with atopic dermatitis based on history of eczema herpeticum infection and Staphylococcus aureus colonization. J Allergy Clin Immunol. (2011) 127:186–93.e181–111. 10.1016/j.jaci.2010.10.033 21211653 PMC3059191

[8] Sanyal RD Pavel AB Glickman J Chan TC Zheng X Zhang N. Atopic dermatitis in African American patients is TH2/TH22-skewed with TH1/TH17 attenuation. Ann Allergy Asthma Immunol. (2018) 122:99–110. 10.1016/j.anai.2018.08.024 30223113

[9] Noda S Suarez-Farinas M Ungar B Kim SJ De Guzman Strong C Xu H. The Asian atopic dermatitis phenotype combines features of atopic dermatitis and psoriasis with increased TH17 polarization. J Allergy Clin Immunol. (2015) 136:1254–64. 10.1016/j.jaci.2015.08.015 26428954

[10] Wen HC Czarnowicki T Noda S Malik K Pavel AB Nakajima S. Serum from Asian patients with atopic dermatitis is characterized by TH2/TH22 activation, which is highly correlated with nonlesional skin measures. J Allergy Clin Immunol. (2018) 142:324–8.e311. 10.1016/j.jaci.2018.02.047 29653116

[11] Zhou L Leonard A Pavel AB Malik K Raja A Glickman J. Age-specific changes in the molecular phenotype of patients with moderate-to-severe atopic dermatitis. J Allergy Clin Immunol. (2019) 144:144–56. 10.1016/j.jaci.2019.01.015 30685456

[12] Hamilton JD Suarez-Farinas M Dhingra N Cardinale I Li X Kostic A. Dupilumab improves the molecular signature in skin of patients with moderate-to-severe atopic dermatitis. J Allergy Clin Immunol. (2014) 134:1293–300. 10.1016/j.jaci.2014.10.013 25482871

[13] Simpson EL Bieber T Guttman-Yassky E Beck LA Blauvelt A Cork MJ. Two phase 3 trials of dupilumab versus placebo in atopic dermatitis. N Engl J Med. (2016) 375:2335–48. 10.1056/NEJMoa1610020 27690741

[14] Simpson EL Paller AS Siegfried EC Boguniewicz M Sher L Gooderham MJ. Efficacy and safety of dupilumab in adolescents with uncontrolled moderate to severe atopic dermatitis: a phase 3 randomized clinical trial. JAMA Dermatol. (2019) 156:44–56. 10.1001/jamadermatol.2019.3336 31693077 PMC6865265

[15] Kim BE Goleva E Kim PS Norquest K Bronchick C Taylor P. Side-by-side comparison of skin biopsies and skin tape stripping highlights abnormal stratum corneum in atopic dermatitis. J Invest Dermatol. (2019) 139:2387–89.e1. 10.1016/j.jid.2019.03.1160 31176708 PMC6814531

[16] Dyjack N Goleva E Rios C Kim BE Bin L Taylor P. Minimally invasive skin tape strip RNA sequencing identifies novel characteristics of the type 2-high atopic dermatitis disease endotype. J Allergy Clin Immunol. (2018) 141:1298–309. 10.1016/j.jaci.2017.10.046 29309794 PMC5892844

[17] Guttman-Yassky E Diaz A Pavel AB Fernandes M Lefferdink R Erickson T. Use of tape strips to detect immune and barrier abnormalities in the skin of children with early-onset atopic dermatitis. JAMA Dermatol. (2019) 155:1358–70. 10.1001/jamadermatol.2019.2983 31596431 PMC6802262

[18] Leung DYM Calatroni A Zaramela LS Lebeau PK Dyjack N Brar K. The nonlesional skin surface distinguishes atopic dermatitis with food allergy as a unique endotype. Sci Transl Med. (2019) 11:eaav2685. 10.1126/scitranslmed.aav2685 30787169 PMC7676854

[19] Berdyshev E Goleva E Bronova I Dyjack N Rios C Jung J. Lipid abnormalities in atopic skin are driven by type 2 cytokines. JCI Insight. (2018) 3:e98006. 10.1172/jci.insight.98006 29467325 PMC5916244

[20] Hulshof L Hack DP Hasnoe QCJ Dontje B Jakasa I Riethmuller C. A minimally invasive tool to study immune response and skin barrier in children with atopic dermatitis. Br J Dermatol. (2019) 180:621–30. 10.1111/bjd.16994 29989151

[21] Mcaleer MA Jakasa I Hurault G Sarvari P Mclean WHI Tanaka RJ. Systemic and stratum corneum biomarkers of severity in infant atopic dermatitis include markers of innate and T helper cell-related immunity and angiogenesis. Br J Dermatol. (2019) 180:586–96. 10.1111/bjd.17088 30132823 PMC6446820

[22] Winget JM Finlay D Mills KJ Huggins T Bascom C Isfort RJ. Quantitative Proteomic analysis of stratum corneum dysfunction in adult chronic atopic dermatitis. J Invest Dermatol. (2016) 136:1732–5. 10.1016/j.jid.2016.03.037 27091361 PMC5018406

[23] Broccardo CJ Mahaffey SB Strand M Reisdorph NA Leung DY. Peeling off the layers: skin taping and a novel proteomics approach to study atopic dermatitis. J Allergy Clin Immunol. (2009) 124:1113–15.e1111. 10.1016/j.jaci.2009.07.057 19748658 PMC8648281

[24] Clausen ML Slotved HC Krogfelt KA Agner T. Measurements of AMPs in stratum corneum of atopic dermatitis and healthy skin-tape stripping technique. Sci Rep. (2018) 8:1666. 10.1038/s41598-018-20204-8 29374283 PMC5786105

[25] Janssens M Van Smeden J Gooris GS Bras W Portale G Caspers PJ. Increase in short-chain ceramides correlates with an altered lipid organization and decreased barrier function in atopic eczema patients. J Lipid Res. (2012) 53:2755–66. 10.1194/jlr.P030338 23024286 PMC3494247

[26] Angelova-Fischer I Mannheimer AC Hinder A Ruether A Franke A Neubert RH. Distinct barrier integrity phenotypes in filaggrin-related atopic eczema following sequential tape stripping and lipid profiling. Exp Dermatol. (2011) 20:351–6. 10.1111/j.1600-0625.2011.01259.x 21410766

[27] Koppes SA Brans R Ljubojevic Hadzavdic S Frings-Dresen MH Rustemeyer T Kezic S. Stratum corneum tape stripping: monitoring of inflammatory mediators in atopic dermatitis patients using topical therapy. Int Arch Allergy Immunol. (2016) 170:187–93. 10.1159/000448400 27584583 PMC5296885

[28] Pavel AB Zhou L Diaz A Ungar B Dan J He H. The proteomic skin profile of moderate-to-severe atopic dermatitis patients shows an inflammatory signature. J Am Acad Dermatol. (2020) 82:690–9. 10.1016/j.jaad.2019.10.039 31669080

[29] Lind L Arnlov J Lindahl B Siegbahn A Sundstrom J Ingelsson E. Use of a proximity extension assay proteomics chip to discover new biomarkers for human atherosclerosis. Atherosclerosis. (2015) 242:205–10. 10.1016/j.atherosclerosis.2015.07.023 26204497

[30] Brunner PM Suárez-Fariñas M He H Malik K Wen HC Gonzalez J. The atopic dermatitis blood signature is characterized by increases in inflammatory and cardiovascular risk proteins. Sci Rep. (2017) 7:8707. 10.1038/s41598-017-09207-z 28821884 PMC5562859

[31] He H Li R Choi S Zhou L Pavel A Estrada YD. Increased cardiovascular and atherosclerosis markers in blood of older patients with atopic dermatitis. Ann Allergy Asthma Immunol. (2020) 124:70–78. 10.1016/j.anai.2019.10.013 31622668

[32] Suarez-Farinas M Tintle SJ Shemer A Chiricozzi A Nograles K Cardinale I. Nonlesional atopic dermatitis skin is characterized by broad terminal differentiation defects and variable immune abnormalities. J Allergy Clin Immunol. (2011) 127:954–64.e951–954. 10.1016/j.jaci.2010.12.1124 21388663 PMC3128983

[33] Lutz MB Schnare M Menges M Rossner S Rollinghoff M Schuler G. Differential functions of IL-4 receptor types I and II for dendritic cell maturation and IL-12 production and their dependency on GM-CSF. J Immunol. (2002) 169:3574–80. 10.4049/jimmunol.169.7.3574 12244147

[34] Tel J Torensma R Figdor CG De Vries IJ. IL-4 and IL-13 alter plasmacytoid dendritic cell responsiveness to CpG DNA and herpes simplex virus-1. J Invest Dermatol. (2011) 131:900–06. 10.1038/jid.2010.410 21191414

[35] Kohli P Bonaca MP Kakkar R Kudinova AY Scirica BM Sabatine MS. Role of ST2 in non-ST-elevation acute coronary syndrome in the MERLIN-TIMI 36 trial. Clin Chem. (2012) 58:257–66. 10.1373/clinchem.2011.173369 22096031 PMC4277435

[36] Gu L Tseng S Horner RM Tam C Loda M Rollins BJ. Control of TH2 polarization by the chemokine monocyte chemoattractant protein-1. Nature. (2000) 404:407–11. 10.1038/35006097 10746730

[37] Lebre MC Burwell T Vieira PL Lora J Coyle AJ Kapsenberg ML. Differential expression of inflammatory chemokines by Th1- and Th2-cell promoting dendritic cells: a role for different mature dendritic cell populations in attracting appropriate effector cells to peripheral sites of inflammation. Immunol Cell Biol. (2005) 83:525–35. 10.1111/j.1440-1711.2005.01365.x 16174103

[38] Kaiko GE Horvat JC Beagley KW Hansbro PM. Immunological decision-making: how does the immune system decide to mount a helper T-cell response? Immunology. (2008) 123:326–38. 10.1111/j.1365-2567.2007.02719.x 17983439 PMC2433332

[39] Magombedze G Eda S Ganusov VV. Competition for antigen between Th1 and Th2 responses determines the timing of the immune response switch during *Mycobaterium avium* subspecies paratuberulosis infection in ruminants. PLoS Comput Biol. (2014) 10:e1003414. 10.1371/journal.pcbi.1003414 24415928 PMC3886887

[40] Brunner PM He H Pavel AB Czarnowicki T Lefferdink R Erickson T. The blood proteomic signature of early-onset pediatric atopic dermatitis shows systemic inflammation and is distinct from adult long-standing disease. J Am Acad Dermatol. (2019) 81:510–19. 10.1016/j.jaad.2019.04.036 31009665

[41] Lisowska A Swiecki P Knapp M Gil M Musial WJ Kaminski K. Insulin-like growth factor-binding protein 7 (IGFBP 7) as a new biomarker in coronary heart disease. Adv Med Sci. (2019) 64:195–201. 10.1016/j.advms.2018.08.017 30769262

[42] Ueland T Akerblom A Ghukasyan T Michelsen AE Becker RC Bertilsson M. ALCAM predicts future cardiovascular death in acute coronary syndromes: insights from the PLATO trial. Atherosclerosis. (2020) 293:35–41. 10.1016/j.atherosclerosis.2019.11.031 31835039

[43] Artieda M Cenarro A Ganan A Jerico I Gonzalvo C Casado JM. Serum chitotriosidase activity is increased in subjects with atherosclerosis disease. Arterioscler Thromb Vasc Biol. (2003) 23:1645–52. 10.1161/01.ATV.0000089329.09061.07 12893688

[44] Sonmez A Haymana C Tapan S Safer U Celebi G Ozturk O. Chitotriosidase activity predicts endothelial dysfunction in type-2 diabetes mellitus. Endocrine. (2010) 37:455–9. 10.1007/s12020-010-9334-4 20960168

[45] Cyrille NB Villablanca PA Ramakrishna H. Soluble urokinase plasminogen activation receptor–An emerging new biomarker of cardiovascular disease and critical illness. Ann Card Anaesth. (2016) 19:214–16. 10.4103/0971-9784.179588 27052059 PMC4900352

[46] Eugen-Olsen J Andersen O Linneberg A Ladelund S Hansen TW Langkilde A. Circulating soluble urokinase plasminogen activator receptor predicts cancer, cardiovascular disease, diabetes and mortality in the general population. J Intern Med. (2010) 268:296–308. 10.1111/j.1365-2796.2010.02252.x 20561148

[47] Kohler HP Grant PJ. Plasminogen-activator inhibitor type 1 and coronary artery disease. N Engl J Med. (2000) 342:1792–801. 10.1056/NEJM200006153422406 10853003

[48] Hamsten A Wiman B De Faire U Blomback M. Increased plasma levels of a rapid inhibitor of tissue plasminogen activator in young survivors of myocardial infarction. N Engl J Med. (1985) 313:1557–63. 10.1056/NEJM198512193132501 3934538

[49] Batlle M Recarte-Pelz P Roig E Castel MA Cardona M Farrero M. AXL receptor tyrosine kinase is increased in patients with heart failure. Int J Cardiol. (2014) 173:402–9. 10.1016/j.ijcard.2014.03.016 24681018

